# Hyperlipemia pancreatitis onset time affects the association between elevated serum triglyceride levels and disease severity

**DOI:** 10.1186/s12944-022-01656-4

**Published:** 2022-05-30

**Authors:** Xiuli Dong, Shuang Pan, Daguan Zhang, Wandong Hong, Tanzhou Chen, Bingxin Zhang, Zhiming Huang, Chengshui Chen

**Affiliations:** 1 Department of Gastroenterology and Hepatology, The First Affiliated Hospital of Wenzhou Medical University, Wenzhou, 325000 Zhejiang China; 2 Department of Respiratory and Critical Care Medicine, The First Affiliated Hospital of Wenzhou Medical University, Key Laboratory of Interventional Pulmonology of Zhejiang Province, Wenzhou, 325000 Zhejiang China

**Keywords:** Acute pancreatitis, Triglyceride, Hypertriglyceridaemia, Severity, Onset

## Abstract

**Background:**

The association of serum triglyceride (TG) levels with the severity of hypertriglyceridaemia-induced acute pancreatitis (HTG-AP) remains controversial. This study aimed to comprehensively assess the TG levels from the initial onset and their predictive value in the disease assessment of HTG-AP.

**Methods:**

Data collected from January 2018 to July 2021 in one institute were assessed retrospectively. HTG-AP was defined as a TG level > 500 mg/dL in the absence of other common aetiologies of AP. The TG levels within 24 hours (24 h), 48 hours (48 h), 3-4 days (3-4 d), and 5-7 days (5-7 d) after symptom onset and their correlations with disease severity in HTG-AP patients were analysed by cross-sectional and longitudinal studies.

**Results:**

In the cross-sectional study, 377 HTG-AP patients were included before lipid-lowering intervention: 216 subjects had their first TG levels measured within 24 h after onset, 91 within 48 h, 50 in 3-4 d, and 20 in 5-7 d. TG levels decreased in the 24 h, 48 h and 3-4 d groups (*P* < 0.001), however, the TG decline in the 5-7 d group had no difference compared with the 3-4 d group. HTG-AP patients with severe or moderately severe disease displayed higher TG levels than those with mild disease in the 24 h and 48 h groups (*P* < 0.050) but not in the 3-4 d or 5-7 d groups. Furthermore, the TG levels were correlated with the modified computed tomography severity index only in the 24 h and 48 h groups, while an association between serum calcium levels and C-reactive protein levels was only present in the 24 h group. Similarly, the TG levels were related to hospital days and ICU days in the 24 h and/or 48 h groups. In the longitudinal study, 165 patients with complete records of TG levels from 24 h to 5-7 d were enrolled. With supportive care and lipid-lowering treatment after admission, the TG levels declined rapidly (*P* < 0.001), and the correlations with disease severity weakened or even disappeared from 24 h to 5-7 d.

**Conclusion:**

TG levels decreased and attenuated the association with disease severity of HTG-AP over the time of onset. The TG levels within the initial 48 h after onset were most useful for the diagnosis and disease assessment of HTG-AP.

## Background

Acute pancreatitis (AP) is the leading cause requiring emergent hospitalization for gastrointestinal diseases. Most AP patients have mild disease (MAP), but approximately 20% of patients progress to moderately severe or severe AP (MSAP or SAP), characteristed with pancreatic tissue necrosis or organ failure, resulting in a 20-40% mortality rate [1, 2].

Hypertriglyceridaemia (HTG) is a common and well-recognized aetiology of AP after gallstones and alcohol consumption [3]. It develops from primary (genetic) and/or secondary lipid metabolism disorders, and the secondary HTG is related to a variety of conditions, including insulin resistance, visceral obesity, alcohol abuse, pregnancy, and hypothyroidism [4]. Although the exact threshold at which HTG induces AP is unkown, generally, a serum TG level > 1000 mg/dL, or the presence of a TG level > 500 mg/dL without other common causes is proposed as the diagnostic criterion for HTG-AP [5–8].

In recent years, the incidence of HTG-AP has increased worldwide, particularly in Asia, rising to 20% of all AP cases in China [9, 10]. Although the definite mechanisms of HTG-AP remain unclear, the prevailing hypothesis is that the excess production of free fatty acids (FFAs), hydrolysed by pancreatic lipase in the presence of elevated TG levels, injures pancreatic acinar cells and capillaries and increases the viscosity of the blood. Eventually, this process causes ischaemia and acidosis of the pancreas, with or without systemic inflammatory response and organ failure [11–17].

HTG-AP has been reported to have a more severe clinical course than AP caused by other conditions [18–22]. Furthermore, many researchers have demonstrated that the increased TG levels also related to the severity of AP, regardless of aetiologies [7, 23–29]. However, others have presented controversial findings [14, 21, 30]. All of these previous studies assessed the association of clinical features and outcome in AP patients with TG levels on admission rather than at the time of symptom onset (e.g., within 24 h, 48 h, or 72 h of admittance or simply picking the first measured value of TG). This is the first study to analyse the correlation of TG levels and clinical severity in HTG-AP according to the same onset times by cross-sectional and longitudinal studies.

## Methods

### Patient inclusion and exclusion criteria

All cases of HTG-AP discharged from the First Affiliated Hospital of Wenzhou Medical University from January 1, 2018 to July 1, 2021 were included in this study. AP was diagnosed by the three manifestations: abdominal pain, elevated blood amylase/lipase level and/or typical radiological appearances [31]. HTG-AP was defined as AP with a TG level > 500 mg/dL and an exclusion of other causes for a more inclusive definition [6]. HTG-AP patients with age < 18 years, pregnancy, pancreatic cancer, or chronic pancreatitis were also excluded. The subjects were categorized into four groups based on the time between the symptom onset to the first measurement of TG levels before any lipid-lowering intervention: 24 h group, 48 h group, 3-4 d group, and 5-7 d group. To obtain a comprehensive view of the TG level changes during the condition’s progression, patients with fully accessible longitudinal TG level data from 24 h to 5-7 d were collected and analysed.

The disease severity of AP inclueds MAP, MSAP and SAP, was categorised by revised Atlanta Classification criteria [31]. Outcomes were assessed by acute pancreatic and/or peripancreatic collections (APCs), pancreatic necrosis, hospitalization days, intensive care unit (ICU) days, organ failure and mortality. Organ failure included respiratory insufficiency (partial pressure of oxygen/fraction of inspired oxygen(PaO2/FiO2) ≤300 mmHg), renal failure (serum creatinine ≥170 μmol/L after rehydration), and/or shock (systolic blood pressure (SBP) ≤90 mmHg) [32, 33].

### Demographic and clinical data collection and ethics

The clinical characteristics, such as age, sex, body mass index (BMI), history of alcohol intake, diabetes mellitus (DM) and fatty liver were retrospectively recorded. Patients with a heavy alcohol drinking history, which was defined as an alcohol intake of ≥40 g/d for ≥5 years, were excluded from this study due to an alcoholic aetiology [22]. Serum calcium (Ca), C-reactive protein (CRP) and white blood cell (WBC) count examined at the same time as the TG level were also collected. TG, serum calcium and CRP were measured by glycerol phosphate oxidase-p-aminophenazone (GPO-PAP) enzymatic method, colorimetry and immune turbidimetric assay, respectively, on a Roche Cobas 8000 c702 analyser. WBC counts were determined with a Mindray BC-6800 haematological analyser.

The modified computed tomography severity index (MCTSI) is based on a 10-point severity scale, which comprises APCs (0-4 points) and the pancreatic necrosis area (0-4 points) plus extrapancreatic complications, including pleural effusion and/or ascites as well as vascular and/or gastrointestinal embolism (0-2 points) [34].

The initial management of HTG-AP patients includes fasting, fluid resuscitation, pain control, and nutritional support [35]. Following the initial management, specific treatments for lowering serum TG were recorded, such as insulin infusion, heparin, plasmapheresis and antihyperlipidaemic drugs [4, 36].

This retrospective and observational study protocol was authorized by the institute’s ethics committee, and patient anonymity was completely protected.

### Statistical methods

Categorical varriable are summarized as frequencies with percentages and analysed by the chi-square test. Continuous varriable are presented as mean with standard deviation (SD) or median with interquartile range (IQR) as appropriate. WBC counts in different groups were compared by one-way ANOVA analysis, while other continuous data, such as TG levels, in patients with different onset time groups and severity subgroups, were assessed by the Kruskal–Wallis nonparametric test, and the Bonferroni test was used for subsequent multiple pairwise comparisons. The correlations of the TG levels with clinical laboratory data, disease severity and outcomes were verified by nonparametric methods (Spearman r). A receiver operating characteristic (ROC) curve was made to check the predictive value of TG levels for MSAP/SAP diseases. The sensitivity, specificity, Youden index, posititive predictive value (PPV) and negative predictive value (NPV) of the cut-off value of TG were analysed by contingency tables method. Statistical analysis was done on SPSS 25.0 software, and *P* < 0.050 was considered as statistical significance.

## Results

### Demographics and clinical features: there were more men and younger individuals and more comorbidities, including diabetes mellitus and fatty liver

**Fig. 1:**
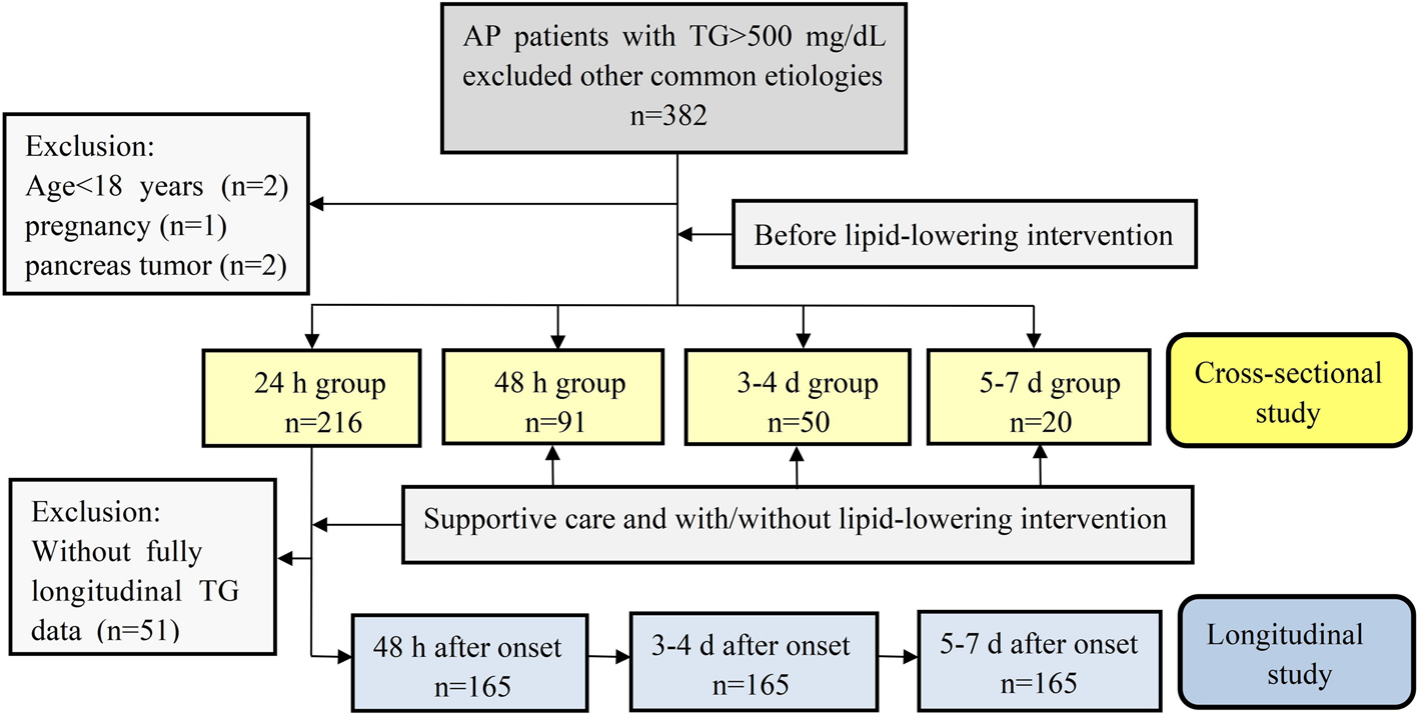
Flow chart of the HTG-AP patient selecttion. The 24 h, 48 h, 3-4 d, and 5-7 d groups were classified by the duration between the time of onset to when the TG levels were first measured. HTG-AP, hyperlipidaemic-induced acute pancreatitis; TG, triglyceride

In total, 377 patients were retrospectively enrolled and classified into 24 h group (*n* = 216), 48 h group (*n* = 91), 3-4 d group (*n* = 50), and 5-7 d group (*n* = 20) based on duration between the time of onset to when the first TG levels were obtained before lipid-lowering treatment. Furthermore, 165 cases in the 24 h group (*n* = 216) had fully available longitudinal TG level data from 24 h to 5-7 d (Fig. 1). Among the 377 patients, 308 (81.7%) were men, the median age was 39.0 (IQR 33.0-45.0) years and the median BMI was 25.8 (IQR 23.7-27.7) kg/m^2^. Among these, 175 (46.4%) patients had a history of alcohol consumption, 41.4% had diabetes, and 89.4% had fatty liver. The difference in sex, age, BMI, alcohol status or comorbidities among the four groups had no significance. There were no deaths due to the inclusion and exclusion criteria of this study. The details of the clinical features are shown in Table 1. All patients received the initial supportive care, among which 182 were treated with heparin and insulin, 119 with insulin infusion alone, 8 with heparin alone, and 10 with a combination of heparin, insulin and plasmapheresis. In addition, 50 patients were subsequently given oral antihyperlipidaemic drugs during their hospitalization.

**Table 1:** Cross-sectional comparison of clinical characteristics and disease severity in HTG-AP patients

Variable	24 h group *N* = 216	48 h group *N* = 91	3-4 d group *N* = 50	5-7 d group *N* = 20	*P* value
Age, median (IQR), years	40.0 (32.5-46.0)	38.0 (34.0-44.0)	38.0 (33.8-45.0)	41.0 (33.3-45.0)	0.818
Male, N (%)	171 (79.2)	79 (86.8)	40 (80.0)	18 (90.0)	0.316
BMI, median (IQR), kg/m^2^	25.8 (23.7-27.9)	25.6 (23.1-28.2)	26.4 (24.6-29.4)	25.4 (23.7-26.3)	0.216
Alcohol history, N (%)	91 (42.1)	47 (51.6)	26 (52.0)	11 (55.0)	0.282
Diabetes mellitus, N (%)	95 (44.0)	37 (40.7)	18 (36.0)	6 (30.0)	0.515
Fatty liver, N (%)	188 (87.0)	83 (91.2)	47 (94.0)	19 (95.0)	0.339
Disease severity, N (%)					
MAP	119 (55.1)	48 (52.7)	31 (62.0)	14 (70.0)	0.457
MSAP	72 (33.3)	29 (31.9)	11 (22.0)	3 (15.0)	
SAP	25 (11.6)	14 (15.4)	8 (16.0)	3 (15.0)	
TG, median (IQR), mg/dL	2425.2(1420.6-4700.0)	1409.7(863.7-2294.7)	853.1(646.5-1008.2)	769.5(598.2-1056.4)	< 0.001
MAP	1970.8 (1123.0-2887.6)	1123.5 (705.1-1593.8)	829.2 (647.8-969.0)	735.0 (539.4-928.3)	
MSAP	3188.5 (2087.2-5892.3)	1705.3 (1099.6-2874.8)	797.3 (543.4-1189.4)	813.3 (604.4-1462.8)	
SAP	5113.3 (2368.6-6739.8)	2047.8 (882.8-2950.9)	998.3 (726.1-1494.5)	1551.1 (564.6-1730.1)	
Ca, median (IQR), mmol/L	2.11 (1.93-2.23)	2.13 (1.86-2.28)	2.18 (2.03-2.29)	2.16 (2.01-2.35)	0.093
CRP, median (IQR), mg/L	19.6(7.2-72.9)	68.5(20.5-145.4)	93.5(54.8-191.2)	81.5(26.8-115.9)	< 0.001
WBC (10^9), mean (SD)	13.86 (4.13)	13.05 (5.10)	14.67 (5.14)	11.76 (3.78)	0.041
MCTSI, median (IQR)	4.0 (2.0-6.0)	4.0(2.0-6.0)	4.0(2.0-6.0)	4.0(2.0-4.0)	0.375
Hospital days, median (IQR)	11.0 (7.0-16.0)	11.0 (8.0-17.0)	9.5 (6.0-15.0)	9.5 (6.3-16.8)	0.615
ICU need, N (%)	64 (29.6)	31(34.1)	14(28.0)	6(30.0)	0.856
ICU free days, median (IQR)	10.0 (7.0-15.0)	11.0 (8.0-16.0)	9.5 (6.0-14.3)	9.5 (6.3-15.5)	0.664

Data are numbers (N) and percentages (%), or mean (SD), medians (IQR, 25th-75th percentile)

*SD* standard deviation, *IQR* interquartile range, *HTG-AP* hypertriglyceridaemia-induced acute pancreatitis, *TG* triglyceride, *MAP/MSAP/SAP* mild/moderately severe/severe acute pancreatitis, *BMI* body mass index, *Ca* calcium, *CRP* C-reactive protein, *WBC* white blood cell, *MCTSI* modified computed tomography severity index, *ICU* intensive care unit

### Cross-sectional study: TG levels significantly decreased over time and attenuated the association with the disease severity in HTG-AP patients based on onset time

**Fig. 2:**
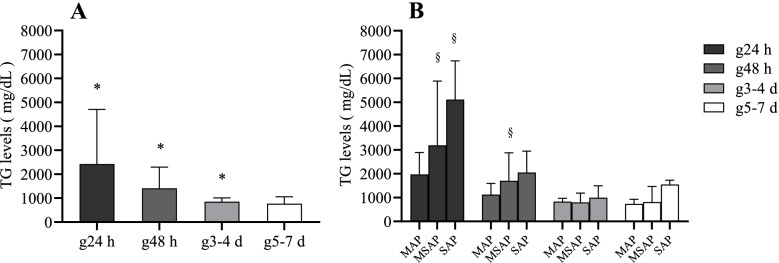
Cross-sectional study: TG levels significantly decreased and attenuated the association with disease severity in the HTG-AP patients. **A** The comparison of TG levels in g24 h, g48 h, g3-4 d, g5-7 d groups. **B** The comparison of TG levels in MAP, MSAP and SAP patients in g24 h, g48 h, g3-4 d, g5-7 d groups respectively. Data are presented as medians (IQR, 25th-75th percentile). g24 h, g48 h, g3-4 d, g5-7 d groups are based on the duration between the time of onset to when first measured TG levels in HTG-AP patients, represent 24 h group, 48 h group, 3-4 d group, and 5-7 d group respectively. HTG-AP, hypertriglyceridaemia-induced acute pancreatitis; TG, triglyceride; MAP/MSAP/SAP, mild/moderately severe/severe AP; IQR, interquartile range. * indicates significance when compared among g24 h, g48 h, g3-4 d *(P* < 0.050). § indicates significance when compared with MAP disease (*P* < 0.050)

The TG levels among the 24 h, 48 h, 3-4 d, and 5-7 d groups had significant difference (*P* < 0.001) (Table 1). The multiple comparisons test showed that TG levels significantly decreased over time after onset in the 24 h group, 48 h group, and 3-4 d group (g24 h vs. g48 h, *P* < 0.001. g24 h vs. g3-4 d, *P* < 0.001. g48 h vs. g3-4 d, *P* = 0.002), but the TG levels in the 5-7 d group did not significantly decline compared with those in the 3-4 d group (*P* = 1.000) (Fig. 2A). The constituent ratio of patients with different severity subgroups had no significant differences (*P* = 0.457) and the TG levels of each subgroup are shown in Table 1. In both the 24 h and 48 h groups, patients with MSAP/SAP disease displayed significantly higher TG levels than MAP patients (MAP vs. MSAP/SAP, both *P* < 0.001 in the 24 h group. MAP vs. MSAP/SAP, *P* = 0.014, *P* = 0.061, respectively, in the 48 h group), but no difference was found between MSAP and SAP patients in either group (*P* = 0.673, *P* = 1.000, respectively). In contrast, there were no differences of TG levels among the three disease severity subgroups in the 3-4 d and 5-7 d groups (*P* = 0.279 and *P* = 0.394, respectively) (Fig. 2B).

The nonparametric correlation analysis indicated that TG levels had significant association with disease severity in both the 24 h and 48 h groups (*r* = 0.387, *P* < 0.001; *r* = 0.329, *P* = 0.001, respectively) but not in the 3-4 d or 5-7 d groups. A negative association between TG levels and serum calcium levels was only presented in the 24 h group (*r* = − 0.502, *P* < 0.001) (Table 2). Although the CRP and WBC levels were different among the four groups (*P* < 0.001, *P* = 0.041, respectively) (Table 1), only the TG levels in the 24 h group had a positive correlation with CRP levels (*r* = 0.193, *P* = 0.004), and no significant correlations were found between TG and WBC in any groups. Similarly, the TG levels were correlated with MCTSI only in the 24 h and 48 h groups (*r* = 0.322, *P* < 0.001; *r* = 0.218, *P* = 0.038, respectively). Moreover, the elevated TG levels were associated with prolonged hospital days in the 24 h and 48 h groups (*r* = 0.349, *P* < 0.001; *r* = 0.281, *P* = 0.007, respectively), while an association with the requirement of ICU days was only observed in the 24 h group (*r* = 0.272, *P* < 0.001) but not in the other groups (Table 2).

**Table 2:** Correlation of TG levels with disease severity and clinical indicators in HTG-AP patients

Correlation (r, *P*)	24 h group TG levels		48 h group TG levels		3-4 d group TG levels		5-7 d group TG levels	
Disease severity	0.387*	< 0.001	0.329*	0.001	0.118	0.413	0.312	0.181
Serum calcium	−0.502*	< 0.001	−0.180	0.087	− 0.213	0.138	− 0.297	0.203
C-reactive protein	0.193*	0.004	0.195	0.064	0.172	0.231	0.182	0.442
White blood cell	−0.116	0.088	0.071	0.071	−0.274	0.054	0.153	0.519
Modified CT severity index (MCTSI)	0.322*	< 0.001	0.218*	0.038	0.152	0.294	0.199	0.399
Hospital days	0.349*	< 0.001	0.281*	0.007	0.021	0.884	0.349	0.132
Intensive care unit (ICU) need days	0.274*	< 0.001	0.200	0.058	0.242	0.090	0.170	0.474

*HTG-AP* hypertriglyceridaemia-induced acute pancreatitis, *TG* triglyceride

^*^P < 0.050

**Fig. 3:**
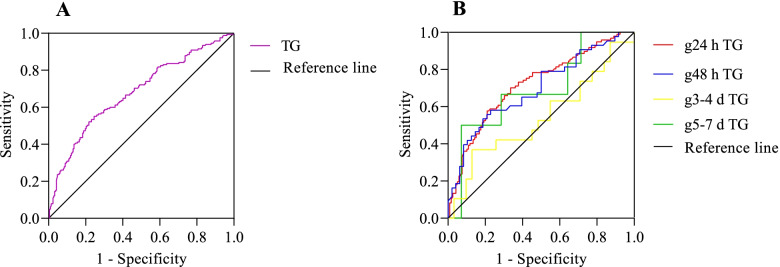
ROC curves of TG levels for predicting MSAP/SAP diseases in HTG-AP patients. **A** ROC curve of TG levels for predicting MSAP/SAP diseases in HTG-AP in all cross-sectional subjects. **B** ROC curves of TG levels for predicting MSAP/SAP diseases in HTG-AP in the different onset time groups. g24 h, g48 h, g3-4 d, g5-7 d groups based on the duration between the time of onset to when the TG levels were first measured in HTG-AP patients. ROC, receiver-operating characteristic; HTG-AP, hypertriglyceridaemia-induced acute panceatitis; TG, triglyceride; MSAP/SAP, moderately severe/severe AP

To further identify the value of TG for the predictive of MSAP or SAP diseases from MAP, an ROC analysis of TG levels was performed on all subjects and different onset time groups. The area under the ROC curve (AUC) for the prediction of MSAP/SAP in all cross-sectional subjects was 0.680 (*P* < 0.001). The AUC was 0.720 (*P* < 0.001) in the 24 h group, and 0.697 (*P* = 0.001) in the 48 h group, however, there was no significance in the 3-4 d group (AUC = 0.542, *P* = 0.624) and 5-7 d group (AUC = 0.698, *P* = 0.187) (Fig. 3, Table 3). The cut-off point for MSAP/SAP of TG was 3054.0 mg/dL with sensitivity of 57.7%, specificity of 79.0%, PPV of 71.3%, and NPV of 67.6% in the 24 h group, and 1625.2 mg/dL with sensitivity of 58.1%, specificity of 77.1%, PPV of 60.9%, and NPV of 75.0% in the 48 h group (Table 3).

**Table 3:** The values of TG levels in the prediction of MSAP/SAP disease in HTG-AP patients

Characteristics	AUC	Std.error	*P*	95% CI Lower bound	95% CI Lower bound	Cut-off points	Sensitivity	Specificity	Youden index	PPV	NPV
TG	0.680	0.028	< 0.001	0.625	0.735	2288.1	0.552	0.693	0.307	0.595	0.655
TG of 24 h group	0.720	0.035	< 0.001	0.651	0.788	3054.0	0.577	0.790	0.367	0.713	0.676
TG of 48 h group	0.697	0.055	0.001	0.588	0.805	1625.2	0.581	0.771	0.352	0.609	0.750
TG of 3-4 d group	0.542	0.088	0.624	0.369	0.714	–	–	–	–	–	–
TG of 5-7 d group	0.698	0.135	0.187	0.426	0.955	–	–	–	–	–	–

*AUC* area under curve, *Std.error* standard deviation error, *95% CI* 95% confidence interval, *PPV/NPV* positive/negative predictive value, *HTG-AP* hypertriglyceridaemia-induced acute pancreatitis, *TG* triglyceride, *MSAP/SAP* moderately severe/severe acute pancreatitis

### Longitudinal study: TG levels remarkably declined, and the correlation with disease severity weakened over time in the HTG-AP patients

**Fig. 4:**
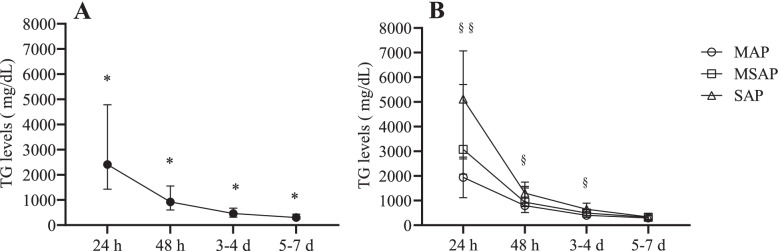
Longitudinal study: TG levels remarkably declined, and the correlation with disease severity weakened over time in the HTG-AP patients. **A** The TG level changed after the onset of symptoms from 24 h to 5-7 d. **B** The difference in TG levels in MAP, MSAP and SAP patients during the onset time of 24 h to 5-7 d. Data are presented as the medians (IQR, 25th-75th percentile). The 24 h, 48 h, 3-4 d, and 5-7 d are based on duration between the time of onset to when the TG levels were measured in HTG-AP patients. TG, triglyceride; HTG-AP, hypertriglyceridaemia-induced acute pancreatitis; MAP/MSAP/SAP, mild/moderately severe/severe AP; IQR, interquartile range. * significance when compared among different onset times (*P* < 0.050). §§ significance for both in MSAP and SAP disease when compared with MAP disease (*P* < 0.050). § significance only for SAP disease when compared with MAP disease (*P* < 0.050)

The fully available longitudinal data of TG levels in 165 cases showed a significant drop over 24 h to 5-7 d with standardized management of pancreatitis and TG levels after hospitalization (*P* < 0.001) (Table 4). Post hoc multiple comparisons tests showed that the differences among all groups were significant (*P* < 0.001) (Fig. 4A). Meanwhile, as shown in Table 4, the percentage of TG levels > 500 mg/dL or > 1000 mg/dL significantly declined over time after onset (both *P* < 0.001). The TG levels fell rapidly below the diagnostic criteria of > 500 mg/dL at 3-4 d and 5-7 d. For the criteria of > 1000 mg/dL, the diagnostic proportion dropped to 46.1% at 48 h and to 9.1% at 3-4 d compared to 0.6% at 5-7 d.

**Table 4:** Longitudinal comparison of TG levels and the correlation with disease severity in HTG-AP patients

Variable	HTG-AP patients, *N* = 165				*P* value
	24 h	48 h	3-4 d	5-7 d	
TG, median (IQR), mg/dL	2408.8 (1428.8-4785.0)	921.2 (604.9-1558.4)	461.9 (315.5-674.4)	302.7 (229.2-434.5)	< 0.001
> 500, N(%)	165 (100)	137 (83.0)	73 (44.2)	28 (17.0)	< 0.001
> 1000,N(%)	148 (89.7)	76 (46.1)	15 (9.1)	1 (0.6)	< 0.001
MAP (*n* = 86)	1943.8 (1117.5-2689.2)	797.4 (513.5-1511.7)	402.7 (289.6-632.5)	294.3 (216.8-404.0)	
MSAP (*n* = 57)	3078.8 (2068.6-5713.3)	938.1 (755.3-1578.8)	494.7 (317.3-650.0)	321.2 (245.1-472.6)	
SAP (*n* = 22)	5118.2 (2764.2-7072.6)	1300.5 (890.2-1752.3)	650.9 (396.5-899.1)	338.1 (230.6-481.9)	
Correlation of TG and disease severity	0.407*	0.209*	0.200*	0.115	

Data are numbers (N or n) and percentages (%), or medians (IQR, 25th-75th percentile)

*IQR* interquartile range, *HTG-AP* hypertriglyceridaemia-induced acute pancreatitis, *TG* triglyceride, *MAP/MSAP/SAP* mild/moderately severe/severe acute pancreatitis

^*^P < 0.050

As shown in Table 4, 86 (52.1%) out of 165 subjects had MAP, 57 (34.5%) had MSAP, and 22 (13.3%) had SAP disease. Significant differences in TG levels among patients with MAP, MSAP, and SAP conditions existed at 24 h, 48 h, and 3-4 d (*P* < 0.001, *P* = 0.026, *P* = 0.024, respectively) but not at 5-7 d (*P* = 0.341). At 24 h, patients with MSAP/SAP disease had higher TG levels than MAP patients (MAP vs. MSAP/SAP, both *P* < 0.001), but no difference existed between the MSAP and SAP (*P* = 0.354). However, at 48 h and 3-4 d, only SAP patients displayed a significantly higher TG level than MAP patients (MAP vs. SAP, *P* = 0.040, MAP vs. MSAP, *P* = 0.241, MSAP vs. SAP, *P* = 0.729 at 48 h; MAP vs. SAP, *P* = 0.023, MAP vs. MSAP, *P* = 0.562, MSAP vs. SAP, *P* = 0.298 at 3-4 d) (Fig. 4B). Further correlation analysis revealed that TG levels were associated with disease severity at 24 h, 48 h, and 3-4 d (*r* = 0.407, *P* < 0.001; *r* = 0.209, *P* = 0.007; *r* = 0.200, *P* = 0.010, respectively) but not at 5-7 d (*r* = 0.115, *P* = 0.143) (Table 4).

## Discussion

The population and clinical data in this study revealed that the majority of HTG-AP patients were male and young. Almost half of them had a drinking history and diabetes mellitus, and nearly 90% had fatty liver, as expected based on previous studies [6, 7, 23–25]. HTG-AP is considered to be a comorbidity of metabolic syndrome. Diabetes mellitus, fatty liver, and obesity are related to the progression and severity of HTG-AP [37]. However, the detailed mechanisms underlying these associations are not clear. Obesity may amplify the inflammatory response of pancreatic and peri-pancreatic fat tissue and fatty liver may attenuate the liver detoxification of inflammatory mediators to aggravate the severity of HTG-AP. The insulin resistance of diabetes may lead to excess FFAs move into the liver to produce more TG. All of these conditions, are likely to increase systemic organ burden and the risk of organ failure [37–39]. Hence, to prevent recurrent disease, the successful management of HTG-AP consists of both pharmacologic therapy and lifestyle modifications. The TG levels should be maintained at < 500 mg/dL or even < 200 mg/dL [3, 40]. Lifestyle modifications include limiting the intake of fat and alcohol, weight loss,and control of fatty liver and diabetes mellitus [3, 4, 32, 36, 41, 42].

Although previous researches have reported that the elevated TG levels are correlated with organ failure and worse outcomes in HTG-AP [7, 23–29] and even dose-dependently associated with the disease severity in AP regardless of the aetiologies [23, 43], other studies have not consistently shown a similar significant correlation [14, 21, 30]. These controversial results may be due to the diverse times at which TG levels were measured in these studies. Most publications measured TG levels within 24 h, 48 h, or 72 h after hospitalization rather than at the onset of symptoms, thus, the definitive course of TG levels is unclear, and the history of lipid-lowering interventions is even less clear.

This is the first study to analyse TG level changes and their association with clinical severity in HTG-AP according to the symptom onset time. Both the cross-sectional and longitudinal data in our study demonstrated that TG levels significantly decreased during the first 3–4 d of disease evolution and continually decreased over 5–7 d after lipid-lowering management. Meanwhile, the fully available longitudinal data showed that TG levels fell rapidly below the diagnostic threshold within 48 h for most patients, and it was extremely difficult to identify HTG-AP based on the diagnostic criteria of > 1000 mg/dL after 3-4 d. The possible mechanism is that the TG-rich chylomicrons in the blood stream is eliminated rapidly in the fasting condition and is further decreased by the initial management of fluid infusion, eventually, a low TG level may be obtained by the subsequent specific HTG treatment modalities [3, 41, 44].

Furthermore, the results suggested that patients with MSAP or SAP disease had significantly higher TG levels than MAP patients during the initial stage, and the correlation between TG levels and disease severity decreased over time and eventually disappeared at 3-4 d or 5-7 d. In addition, the elevated TG levels in the cross-sectional data were associated with MCTSI, serum calcium levels, and CRP only within the initial 24 h to 48 h after onset, and the associations with prolonged hospital stay and the need for an ICU stay tended to be similar over time. An additional ROC curve analysis showed that the AUC in the 24 h group was 0.720 (*P* < 0.001), and a TG level of 3054.0 mg/dL was an accurate threshold for predict MSAP/SAP disease. However, the AUC value decreased in the 48 h group, and there was no significance in the 3-4 d or 5-7 d groups. Given the lack of a significant difference in TG levels between MSAP and SAP diseases in our data, we performed ROC curve analysis to differentiate MSAP/SAP from MAP.

All of these results demonstrated that the correlations between TG levels, disease severity and outcomes in HTG-AP were affected by the time elapsed since the onset of symptoms. During the initial phase, the severity of HTG-AP was associated with the accumulation of FFAs hydrolysed from excess TG, which triggered an inflammatory reaction and had a direct cytotoxic effect on the pancreatic acinar and vascular endothelium. The association was attenuated over time, which may be explained by the natural elimination of TG or FFAs, or subsequent interventions [17]. Calcium was also previously reported to have an negative association with HTG and the severity of AP. The proposed mechanism was that the lipolysis of adipose tissue and the release of FFAs may contribute to hypocalcaemia by the local formation of calcium salts, FFA-albumin complexes binding calcium and damage of the parathyroid function [45–47].

Therefore, TG levels should be measured as early as possible after onset when HTG-AP is suspected. A delayed first visit to the hospital or delayed detection of TG levels after visiting, especially after HTG intervention, will result in a delayed or missed diagnosis of HTG-AP, and a weakened or disappeared predictive value for the disease assessment. However, this may not be suitable during the extremely early stage, as TG levels may be low or normal within fewer than 3 to 6 hours, then peak within 24 h, going down again within 48 h. Another publication proposed that the elevation of TG in AP was considered an epiphenomenon of hydrolysis of visceral adipose tissue by lipase [11].

Given the role of FFAs in the pathophysiology of HTG-AP, a timely reduction of serum TG is commonly considered helpful, since it may be related to a lower risk of development of organ failure and worse outcomes [26]. Multiple therapeutic modalities have been suggested to reduce serum TG levels, such as insulin, heparin, blood purification, and antihyperlipidaemic drugs. However, the benefits of these interventions to the overall outcome of HTG-AP are controversial. More randomized clinical trials would contribute to proposing a generalized and effective treatment modality for HTG-AP in the future [26, 42, 48, 49].

### Study strengths and limitations

The strengths of this study include: 1) it is the first study to comprehensively assess TG level changes tracked from the initial symptom representation and analyse its association with clinical severity in HTG-AP according to different onset times. 2) The results of this study explain the previous controversial studies on the associations between TG levels and the severity of HTG-AP according to hospitalization time, and the TG level within the first 48 h after symptom onset, especially 24 h before any intervention, offers a higher diagnostic rate for HTG-AP and is a more useful biomarker for disease severity assessment.

However, the present study has some limitations, including 1) due to the small sample size in the 5-7 d group in the cross-sectional data, the results from this group are not fully convincing. This may be partly because AP patients usually visit the emergency department in a timely manner with intervention after the onset of symptoms and partly because the TG levels have been reduced to levels lower than the diagnostic criteria by 5-7 d. 2) In the longitudinal study, because it was a retrospective and observational study, all subjects received supportive treatment with or without specific treatment for HTG lipid lowering. These interventions were performed according to the clinical guidelines and had no strictly uniform criteria. A prospective study should therefore be performed with no or uniform lipid lowering intervention to compensate for this defect in future studies.

## Conclusions

This study demonstrated that the TG level decreased and that its association with disease severity and outcomes was attenuated in HTG-AP with the passage of time. The results suggest that it is crucial to determine TG levels within the first 48 h after initial onset, especially 24 h before any intervention in clinical AP patients, which will contribute to avoiding missed diagnosis of HTG-AP and better prognostic evaluation of clinical disease. A prospectively designed multicentre study with large sample size, routine blood lipid testing immediately after admission, and subsequently unified and standardized management will further verify the conclusions of this study and will have a more meaningful guiding role in clinical practice.

## Data Availability

It is available upon reasonable request.

## Abbreviations

AP: Acute pancreatitis
TG: Triglyceride
HTG: Hypertriglyceridaemia
HTG-AP: Hypertriglyceridaemia-induced acute pancreatitis
MAP/MSAP/SAP: Mild/moderately severe/severe acute pancreatitis
FFAs: Free fatty acids
ICU: Intensive care unit
BMI: Body mass index
DM: Diabetes mellitus
Ca: Calcium
CRP: C-reactive protein
WBC: White blood cell
MCTSI: Modified computed tomograph severity index
APCs: Acute pancreatic and/or peripancreatic collections
SD: Standard deviation
IQR: Interquartile range
ROC: Receiver operating characteristic
AUC: Area under the ROC curve
PPV: Positive predictive value
NPV: Negative predictive value
